# Quantitative STAU2 measurement in lymphocytes for breast cancer risk assessment

**DOI:** 10.1038/s41598-020-79622-2

**Published:** 2021-01-13

**Authors:** Charoenchai Puttipanyalears, Sikrit Denariyakoon, Phonthep Angsuwatcharakon, Vitavat Aksornkitti, Mawin Vongsaisuwan, Sutasinee Asayut, Somchai Thanasitthichai, Narisorn Kongruttanachok, Chatchawit Aporntewan, Apiwat Mutirangura

**Affiliations:** 1 Center of Excellence in Molecular Genetics of Cancer and Human Diseases, Department of Anatomy, Faculty of Medicine, Chulalongkorn University, 1873 Rama IV Road, Pathumwan, Bangkok, 10330 Thailand; 2 The Queen Sirikit Center for Breast Cancer, King Chulalongkorn Memorial Hospital, The Thai Red Cross Society, Bangkok, 10330 Thailand; 3 Department of Surgery, Faculty of Medicine, Chulalongkorn University, Bangkok, 10330 Thailand; 4 Research and Technology Assessment Department, National Cancer Institute, Bangkok, 10400 Thailand; 5 Department of Laboratory Medicine, Faculty of Medicine, Chulalongkorn University, Bangkok, 10330 Thailand; 6 Department of Mathematics and Computer Science, Faculty of Science, Chulalongkorn University, Bangkok, 10330 Thailand

**Keywords:** Breast cancer, Cancer genetics, Tumour biomarkers, Cancer, Biomarkers, Cancer genetics, Epigenetics, Gene expression, Genetic markers, Medical genetics

## Abstract

Although mammograms play a key role in early breast cancer detection, the test is not applicable to all women, for example, women under the age of 40. The development of a noninvasive blood test with high sensitivity and accessibility will improve the effectiveness of breast cancer screening programmes. Secretory factors released from cancer cells can induce the expression of certain genes in a large number of white blood cells (WBCs). Therefore, cancer-dependent proteins in WBCs can be used as tumour markers with high sensitivity. Five proteins (LMAN1, AZI2, STAU2, MMP9 and PLOD1) from a systemic analysis of a variety of array data of breast cancer patients were subjected to immunofluorescence staining to evaluate the presence of fixed WBCs on 96-well plates from 363 healthy females and 358 female breast cancer patients. The results revealed that the average fluorescence intensity of anti-STAU2 and the percentage of STAU2-positive T and B lymphocytes in breast cancer patients (110.50 ± 23.38 and 61.87 ± 12.44, respectively) were significantly increased compared with those in healthy females (56.47 ± 32.03 and 33.02 ± 18.10, respectively) (*p* = 3.56 × 10^–71^, odds ratio = 24.59, 95% CI = 16.64–36.34). The effect of secreted molecules from breast cancer cells was proven by the increase in STAU2 intensity in PBMCs cocultured with MCF-7 and T47D cells at 48 h (*p* = 0.0289). The test demonstrated 98.32%, 82.96%, and 48.32% sensitivity and 56.47%, 83.47%, and 98.62% specificity in correlation with the percentage of STAU2-positive cells at 40, 53.34 and 63.38, respectively. We also demonstrated how to use the STAU2 test for the assessment of risk in women under the age of 40. STAU2 is a novel breast cancer marker that can be assessed by quantitative immunofluorescence staining of fixed WBCs that are transportable at room temperature via mail, representing a useful risk assessment tool for women without access to mammograms.

## Introduction

A highly sensitive and specific biomarker has benefited a number of clinical applications, especially cancer screening and treatment monitoring^1–3^. The early detection of breast cancer using an effective approach in cancer screening will contribute to successful treatment and reduce the mortality rate, disease burden and treatment expense^4,5^. Breast cancer is the most common cancer in females worldwide^6^. Currently, mammography is a widely available population-based technique used to detect breast cancer at an early stage and has been proven to decrease the mortality rate^7^. Routine mammography provides 68–85% sensitivity and 72–86% specificity, but inconsistencies in sensitivity and specificity have been reported^8,9^. The sensitivity is considerably reduced in highly dense breasts by 50%^10^. The most effective but expensive techniques for breast cancer diagnosis are breast magnetic resonance imaging (MRI), which is also known as MRI mammography and exhibits > 92% sensitivity and specificity^11,12^. However, one study indicated that the sensitivity of MRI mammography is decreased in women with high breast density^13^. To date, breast MRI is recommended as an adjunct to mammograms in the case of screening high genetic risk individuals.

Mammography screening has been suboptimally accessible in many populations and even in a population of female healthcare workers^14^. Moreover, awareness of radiation exposure limits mammogram screening in young women (below 40) even though the risk of breast cancer is 0.5%^15^. There are a number of barriers that prevent females worldwide from accessing the current breast cancer screening programme. First, the female must be exposed to radiation. Second, the test is painful, causing screening avoidance. Third, many facilities are required, i.e., machines and mammogram readers, making accessibility to mammogram testing difficult^16–19^.

Thus, many researchers focus on the development of tumour markers in blood samples as a less invasive and easy-to-access approach^20–23^. Unfortunately, most current circulating breast cancer markers, including serum tumour markers (CA15-3, CA27.29, CEA), circulating cancer DNA or cell-free DNA (mutation of TP53, KRAS, PIK3CA and promoter methylation of APC, BRCA1, TWIST) and circulating tumour cells (CTCs), exhibit low sensitivity^24–26^.

Recent studies have demonstrated that changes in white blood cells (WBCs) of cancer patients exhibit promising sensitivity^27–30^. Our previous study found that breast cancer cells can secrete factors to regulate gene expression profiles and epigenetic changes in surrounding WBCs, especially gene-containing long interspersed element 1 (LINE-1)^27^. Mucin 1 (MUC-1)-positive plasma cells were identified as a potential marker in the micrometastatic lymph nodes of breast cancer patients with high efficiency. This study will apply the knowledge of secreted factors on induced WBC changes in blood circulation as a cancer-screening tool (Supplementary data 1). In addition, we previously demonstrated that LINE-1 hypermethylation and upregulation of genes containing LINE-1 are unique characteristics of breast cancer WBCs^27,31^. Therefore, to select unique breast cancer markers in WBCs, we concentrated on genes containing LINE-1. For future screening or diagnosis purposes, upregulated genes were selected. We disregarded downregulated genes because it is more practical to determine the presence of a marker or its increased levels in patients compared with its absence or decreased levels.

Accessibility is the key to success in nationwide screening programmes. The ability to transfer samples by mail is a well-known effective approach for mass screening for inborn errors of metabolism^32^. Here, we tested for breast cancer-inducing proteins in fixed WBCs. Samples prepared by this method are stable and transportable by mail.

## Materials and methods

### Bioinformatics approach (CU-DREAM and CU-DREAM extra)

First, three Gene Expression Omnibus (GEO) datasets were collected from the National Center for Biotechnology Information (NCBI) database, including GSE9014^33^, GSE31138 and GSE27562^34^. The GSE9014 gene expression microarray, which included stromal cells and invasive breast cancer tissues, was compared with the list of genes containing LINE-1. GEO datasets were analysed for the expression levels of genes containing LINE-1 using the CU-DREAM-extra (Connection Up- or Down- Regulation Expression Analysis of Microarrays Extra, website: http://pioneer.netserv.chula.ac) program^35^ (1462 genes containing LINE-1 in the library), and *p* values and odds ratios were calculated. GSE31138 and GSE27562 gene expression microarrays that assessed healthy female WBCs and breast cancer WBCs were compared. GEO datasets were analysed for gene expression levels using the CU-DREAM (Connection Up- or Downregulation Expression Analysis of Microarrays, website: http://pioneer.netserv.chula.ac) program, which calculates *p* values and odds ratios. All upregulated genes containing LINE-1 from GSE9014 and upregulated genes from GSE31138 & GSE27562 were analysed, and protein expression in each candidate gene was compared using the Protein Atlas database (https://www.proteinatlas.org/). We manually observed the data from the Protein Atlas compared with the significantly upregulated genes. The protein expression levels of the 14 upregulated genes (Supplement data 3) obtained from intersecting data were evaluated in the Protein Atlas. Five candidate genes with significant *p* values and moderate levels of protein expression in tissue sections of the Protein Atlas database were selected from the list of upregulated genes to observe gene expression by immunofluorescence staining in blood samples.

### Blood sample collection

Two millilitres of ethylene diamine (EDTA) blood samples were collected from 358 breast cancer patients and 363 healthy females from September 2016 to March 2018. All cancer cases were staged according to the revised TNM classification criteria^36^ by a pathologist. All healthy females were recruited from patients without immune disorders, chronic diseases, and family history of cancer and negative mammography examination results. The mammogram results of all participants were graded by a radiologist. EDTA blood samples were provided by the National Cancer Institute, Thailand and King Chulalongkorn Memorial Hospital. All samples were centrifuged for 15 min at 3000 rpm to separate the WBC layer (buffy coat). Then, 100 µl of the WBC layer was transferred to a 1.5-ml Eppendorf tube. All subjects participating in blood collection were given a self-administered questionnaire to collect their medical history, which was carefully recorded. All samples were obtained under a protocol approved by the Ethics Committee, Faculty of Medicine, Chulalongkorn University, Thailand (approval number: IRB 034/59), and National Cancer Institute, Thailand (approval number: 157_2016RC_OUT487). Thai Clinical Trials Registry number is TCTR20180804002. The collection of blood samples from all participants was performed based on the WHO guidelines. This study was conducted in accordance with the Declaration of Helsinki. Supplementary data 2 provides the STROBE checklist. Signed informed consent was obtained.

### Peripheral blood mononuclear cells (PBMCs) separation

The PBMC fraction, including lymphocytes and monocytes, was separated by standard Ficoll-Hypaque gradient centrifugation following the manufacturer’s protocol (Amersham Pharmacia, Uppsala, Sweden). Briefly, 5 ml of Ficoll-Hypaque gradient was prepared in 15-ml centrifuge tubes. EDTA blood was diluted 1:1 in phosphate-buffered saline (PBS) and carefully layered over a Ficoll-Hypaque gradient (9–10 ml/tube). The sample tubes were centrifuged for 20 min at 1000*g*. The cells in the upper layer were carefully collected and washed twice with PBS for 10 min at 650 g. The cells were resuspended in RPMI 1640 medium with GlutaMAX supplemented with penicillin (50 U/ml)-streptomycin (50 g/ml) and 10 mM HEPES (complete RPMI medium) before counting.

### Cell lines and coculture conditions

In this study, the human carcinoma cell lines, which were provided by the American Type Culture Collection (ATCC, VA, USA), included two mammary carcinoma cell lines: MCF-7 [ATCC-HTB22, ER+ , PR+ , HER2−] and T47D [ATCC-HTB133, ER+ , PR+ , HER2+]. The cell lines were cultured with DMEM supplemented with 10% FBS (Gibco BRL, Life Technologies) at 37 °C in a humidified atmosphere (95% air: 5% CO_2_). The medium was changed every three days, and cell lines were subcultured twice a week. All cell lines were mycoplasma free as determined using the Boehringer Mannheim BM-Cyclin test (F. Hoffmann-La Roche Ltd.). The cells grown in culture flasks (CytoOne T225 flask, USA Scientific Inc.) were collected at 60–65% confluence using 0.05% trypsin and 0.5 mM EDTA and washed in PBS.

The coculture experiments between cancer cells and PBMCs were performed in Transwell culture plates (Costar, Dutscher, Brumath, France) to allow secretory molecule exchange between both cell types as previously described^24^. Breast cancer cell lines were seeded in 24-well culture plates (5 × 10^4^ cells/well) and permitted to attach to serum-free DMEM overnight. Next, the PBMCs were seeded in permanent membrane culture inserts that were 6.5 mm in diameter, and the tissue culture-treated polycarbonate membranes had a 0.4-mm pore size (1 × 10^5^ cells/well). Culture inserts containing PBMCs were placed in wells containing breast cancer cells. PBMCs were harvested, and immunofluorescence staining was performed after cocultivation for 16, 24, and 48 h.

### Immunofluorescence staining

Five microlitres of buffy coat from all blood samples were smeared in 96-well plates. The cells were dried at room temperature for 20 min. Next, 100 µl of 3.7% formaldehyde in PBS was added to the sample for 20 min at room temperature to fix WBCs to assess morphology. The cells were washed 5 times with 100 µl of PBS. The cells were permeabilized with 2% Triton X solution for 10 min at room temperature. All antibodies, including 5 primary antibodies for WBCs (anti-CD45^+^ primary mouse antibody (ab8216) for leukocytes, anti-CD15^+^ primary mouse antibody (ab20137) for polymorphonuclear cells (neutrophils, eosinophils and basophils), anti-CD3^+^ primary mouse antibody (ab8671) for T lymphocytes, anti-CD19 + primary mouse antibody (ab31947) for B lymphocytes, and anti-CD14^+^ primary mouse antibody (ab182032) for monocytes), 5 primary antibodies for candidate proteins (anti-STAU2 primary rabbit antibody (ab184009), anti-AZI2 primary rabbit antibody (ab232654), anti-LMAN1 primary rabbit antibody (ab125006), anti-MMP9 primary rabbit antibody (ab38898) and anti-PLOD1 primary rabbit antibody (ab2647)) and 2 secondary antibodies (goat anti-mouse secondary antibody-Cy3 (ab97035) and goat anti-rabbit secondary antibody-FITC (ab6717)) (Abcam Co., Ltd., Cambridge, England) were prepared in 1:1000 dilution with binding buffer (2% FBS and 0.5% Tween20 in PBS).The primary antibodies were incubated with samples overnight at 37 °C, and the secondary antibodies were incubated for 3 h at 37 °C. The cells were washed 5 times with PBS. Finally, Hoechst nuclear stain (1 µg/µl) was added to the cells and incubated for 15 min at 37 °C. The fluorescent signal from the cells was observed with a confocal microscope (20×).

The fluorescent signals were detected in 3 colours, including blue (Hoechst, 510–540 nm), red (Cy3, 560–570 nm) and green (FITC, 500–520 nm), using a motorized fluorescence microscope type IX83 (Olympus Co., Ltd., USA). The protocol for signal intensity detection and the number of positive cells was operated by CellSens imaging software (Olympus Co., Ltd., USA). Briefly, 12 fields of 20× objective lenses (4 columns × 3 rows) at the centre of each well were captured with specific exposure times as follows: Hoechst (25.5 ms), Cy3 (700 ms) and FITC (316 ms). The signals with 25–200 µm perimeter and 140–300 mean grey intensity values were counted and exported to apply the CancerScreen.exe program (Please contact author for program requirement), which was developed by Aporntewan C. The positive signal in each colour was identified by CancerScreen.exe based on 2 criteria, including the range of perimeter (25–150 µm) and mean grey intensity value in pixels/cell volume units (150–280). The positive cells showed red and green signals in the same position, and the negative cells showed only red signals. Then, the average fluorescence intensity of anti-STAU2 in each sample was calculated from the intensity of positive cells divided by the intensity of positive and negative cells. Moreover, the percentage of STAU2-positive cells was calculated by the number of positive cells divided by the total number of cells. Positive samples from cocultured PBMCs were applied as interassay variation adjustments.

### Statistical analysis and risk assessment

Statistical analyses were performed using SPSS (Statistical Package for the Social Sciences) software for Windows version 17.0.1 (SPSS Inc., Chicago, IL). Data are expressed as the average ± SD, and independent sample t-tests were performed to calculate significant differences among all sample groups. All *p* values are two-sided, and *p* values less than 0.05 are considered statistically significant. Receiver operating characteristic (ROC) curve analysis was performed using MedCalc (Statistical software) for Windows version 11.3.0.0 (Ostend, Belgium). Risk assessment was evaluated by the posterior probability calculation based on Bayes’ theorem.

### Ethics statement

All samples were obtained under a protocol approved by the Ethics Committee, Faculty of Medicine, Chulalongkorn University, Thailand (approval number: IRB 034/59) and the National Cancer Institute, Thailand (approval number: 157_2016RC_OUT487). The collection of blood samples from all participants was performed in accordance with the WHO guidelines. This study was conducted in accordance with the Declaration of Helsinki. The participants provided their written informed consent to participate in this study.

## Results

### Bioinformatics analysis

The GEO datasets were extracted from the NCBI database. The datasets included expression microarrays from breast cancer stromal cells (GSE9014) and breast cancer blood samples (GSE31138 and GSE27562). The intersection results between GSE9014 and the list of genes containing LINE-1 were analysed by CU-DREAM extra, which revealed 709 significantly upregulated genes (*p* = 1.34 × 10^–08^, odds ratio = 1.39). The intersection results between GSE31138 and GSE27562 were analysed by CU-DREAM, which revealed 262 significantly upregulated genes (*p* = 2.62 × 10^–65^, odds ratio = 3.64). The lists of upregulated genes from both intersection results were analysed, and 14 commonly upregulated genes were identified: DENND1B, STAU2, CAMTA1, LPP, PLEKHB2, MTMR2, LMAN1, PHACTR2, EPHA3, COL8A1, DTNBP1, AZI2, MMP9 and PLOD1 (Supplementary data 3). The 14 upregulated genes in breast cancer lymph nodes were assessed using the tissue microarray database from the Protein Atlas (https://www.proteinatlas.org). Then, five upregulated genes with the highest *p* value and positive staining results in the tissue section of the Protein Atlas database were selected as candidate genes to perform immunofluorescence staining for LMAN1, AZI2, STAU2, MMP9 and PLOD1. Only STAU2 exhibited positive results for application as a breast cancer screening marker. The immunofluorescence staining results of other candidate genes are presented in Supplementary data 4.

### Immunofluorescence staining

**Figure 1:**
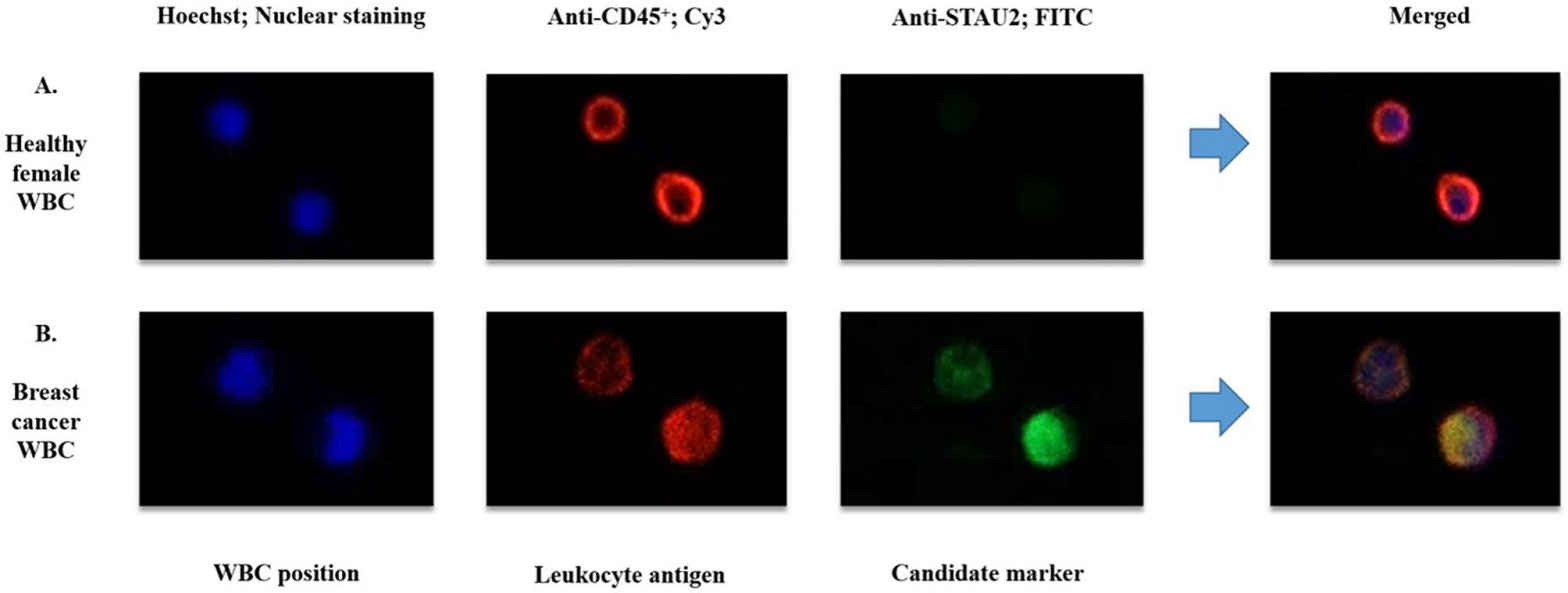
Immunofluorescence signal from WBCs from (**A**) breast cancer patients and (**B**) healthy females observed by confocal microscopy (20×). Hoechst was applied to locate WBC position. Anti-CD45^+^ was used as a positive control for WBCs. The merged group refers to the combination of 3 fluorescent signals. The positive fluorescent signal from anti-STAU2 was exclusively observed in breast cancer WBCs.

WBCs from breast cancer patients (N = 358) and healthy females (N = 363) were subjected to immunofluorescence staining. The demographic data of all patients are presented in Table 1. The following 3 fluorescent signals were observed: (1) Hoechst (blue) is a nuclear stain used to identify the WBC position; (2) anti-CD45^+^ (red) binds to a CD45^+^ molecule, which is a general protein expressed in WBCs; and (3) anti-STAU2 (green) is an antibody that binds to a candidate protein marker for breast cancer screening. Anti-CD45^+^ (red) was observed in WBCs from both breast cancer patients and healthy females (Fig. 1A). The fluorescent signals from anti-STAU2 were indicated in WBCs from breast cancer patients. (Fig. 1B).

**Table 1:** The demographic data of all samples in this study included normal female WBCs (N = 363) from mammograms 1 to 3 and breast cancer WBCs (N = 358) from stage 1 to 4 disease.

Sample groups	N	Age (Average ± SD)	Fluorescent intensity (Average ± SD)	STAU2 positive cells (Average ± SD)*
**Normal female (T and B cells)**	363	61.20 ± 14.14	56.47 ± 32.03	33.02 ± 18.10
Mammogram 1	197	61.30 ± 19.29	53.44 ± 30.96	32.03 ± 18.00
Mammogram 2	118	63.45 ± 14.80	62.81 ± 33.11	36.56 ± 17.66
Mammogram 3	48	58.33 ± 4.09	53.30 ± 32.04	28.34 ± 18.46
**Breast cancer (T and B cells)**	358	61.47 ± 13.09	110.50 ± 23.38	61.87 ± 12.44
Stage 1	189	65.63 ± 17.10	109.51 ± 21.50	61.94 ± 11.96
Stage 2	102	62.50 ± 13.62	108.07 ± 25.47	61.81 ± 13.30
Stage 3	47	55.86 ± 5.08	111.34 ± 22.98	62.24 ± 12.58
Stage 4	19	60.60 ± 13.45	131.24 ± 22.31	59.83 ± 13.39

The average fluorescence intensity and the percentage of STAU2-positive cells are presented as the average ± SD.

Abbreviation: STAU2, staufen-2.

*Data are presented as %.

### Secretory effect in coculture model

**Figure 2:**
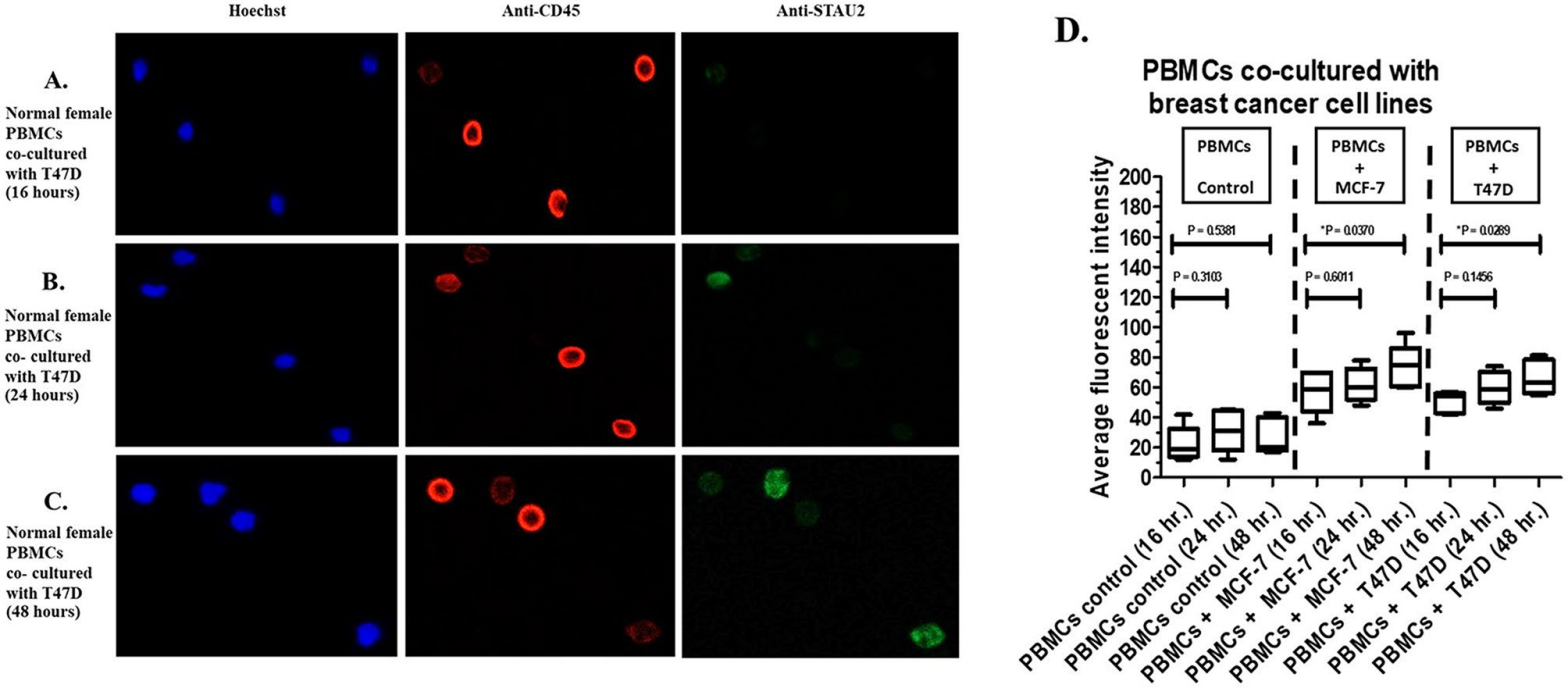
Fluorescent signals were increased in PBMCs cocultured with breast cancer cells. (**A**) The fluorescent signal from PBMCs cocultured with T47D cells at 16 h. (**B**) The fluorescent signal from PBMCs cocultured with T47D cells at 24 h. (**C**) The fluorescent signal from PBMCs cocultured with T47D cells at 48 h. (**D**) An increase in the intensity of the anti-STAU2 fluorescent signal was observed in PBMCs cocultured with both MCF-7 (*p* = 0.0370) and T47D (*p* = 0.0289) cells compared with control PBMCs. The intensity detection was operated by CellSens imaging software (Olympus Co., Ltd., USA).

To observe the effect of secretory molecule release from breast cancer cells, PBMCs were obtained from normal females (N = 5) and cocultured with 2 breast cancer cell lines, MCF-7 and T47D. The average fluorescence intensity of anti-STAU2 from PBMCs cocultured with T47D cells (Fig. 2A–C) was significantly increased at 16, 24, and 48 h (*p* = 0.0289). Increased anti-STAU2 fluorescence intensity was observed in PBMCs cocultured with both MCF-7 and T47D cells (Fig. 2D).

### Cell subtypes

**Figure 3:**
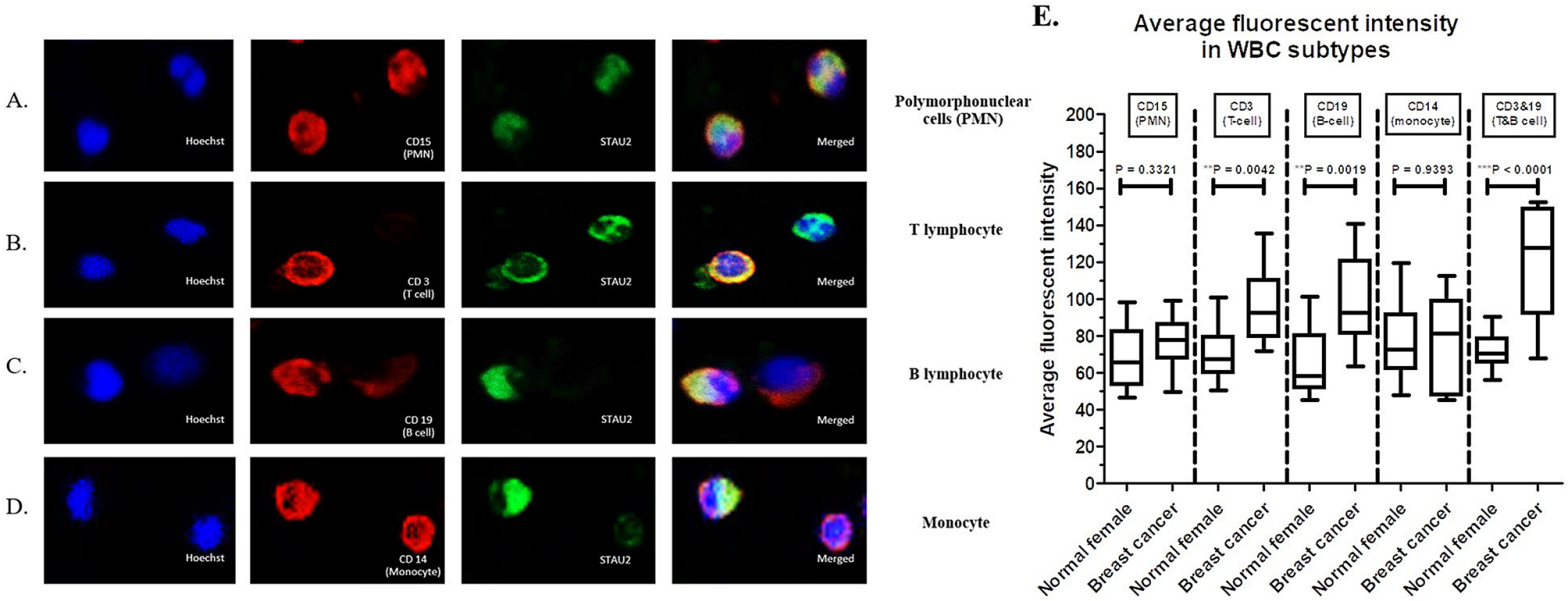
Subtypes of WBCs from breast cancer patients were identified based on specific antibodies, including (**A**) anti-CD15^+^ for polymorphonuclear cells (PMNs); (**B**) anti-CD3^+^ for T lymphocytes; (**C**) anti-CD19^+^ for B lymphocytes; and (**D**) anti-CD14^+^ for monocytes. A positive fluorescent signal was observed in all WBC subtypes. (**E**) The intensity of the anti-STAU2 fluorescent signal was significantly increased in CD3^+^ T-cells (*p* = 0.0042) and CD19^+^ B-cells (*p* = 0.0019). The combination of CD3^+^ and CD19^+^, which refers to the group of T and B lymphocytes, exhibited a notable increase in the average fluorescence intensity (*p* < 0.0001). The intensity detection was operated by CellSens imaging software (Olympus Co., Ltd., USA).

**Figure 4:**
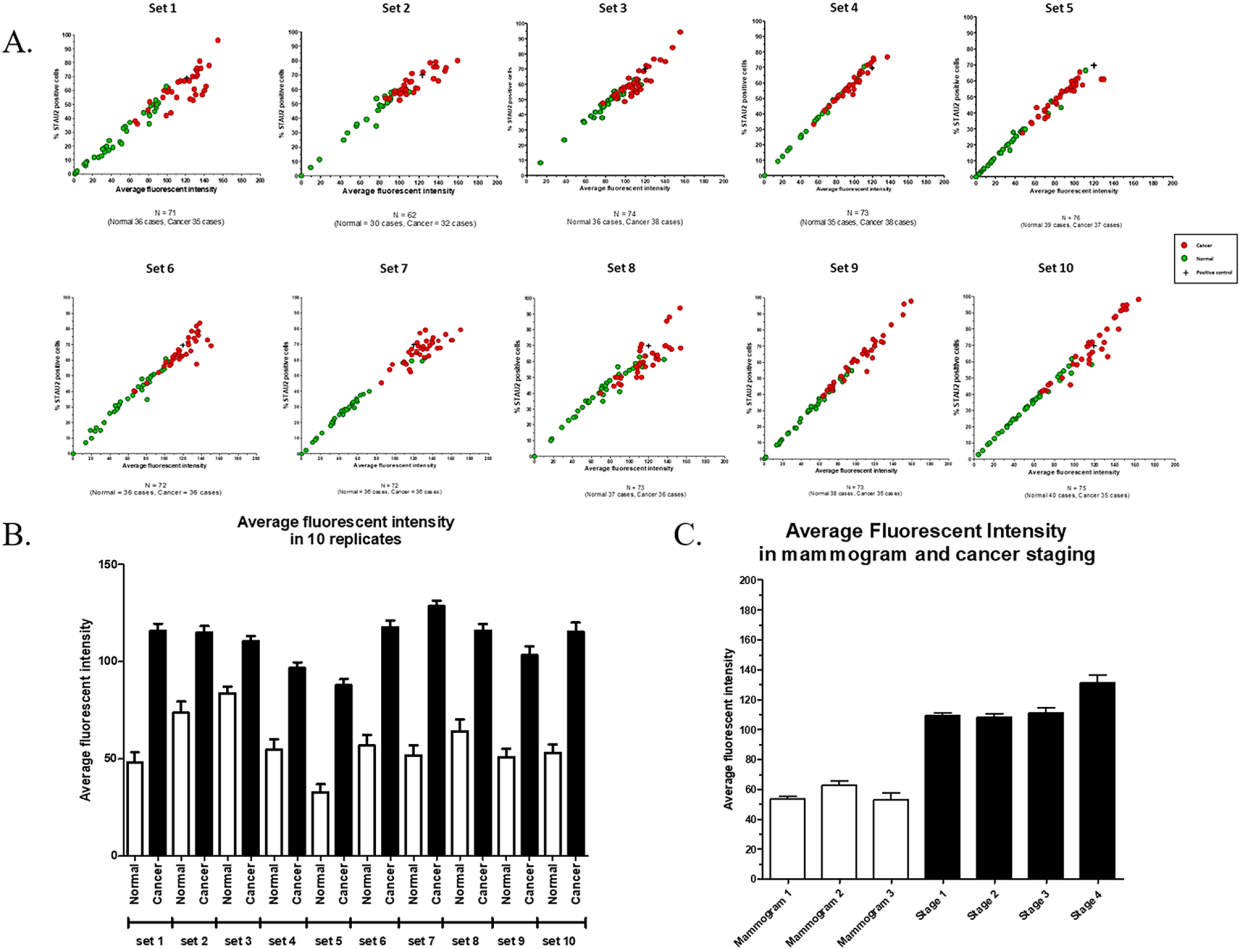
The results of the STAU2 fluorescent signal are presented as the average fluorescence intensity and the percentage of positive cells. (**A**) The replicative test of STAU2 staining, which was performed with a double-blinded test including 10 replicates (sets 1 (N = 71), 2 (N = 62), 3 (N = 74), 4 (N = 73), 5 (N = 76), 6 (N = 72), 7 (N = 72), 8 (N = 73), 9 (N = 73), 10 (N = 75)). Positive samples from cocultured PBMCs were applied as interassay variation adjustments. The average fluorescence intensity and the percentage of STAU2-positive cells exhibited a linear correlation (R^2^ > 0.9). (**B**) Increases in fluorescence intensity and the percentage of positive cells were observed in all replicates (*p* < 0.0001). (**C**) The average fluorescence intensity was not significantly different among WBCs obtained from normal females with 1 to 3 mammograms, but an increasing trend was found from early-stage to late-stage breast cancer patients (*p* = 0.0008). The intensity detection and the percentage of STAU2-positive cells were operated by CellSens imaging software (Olympus Co., Ltd., USA).

Next, four WBC subtypes from breast cancer patients were identified using specific antibodies, including (1) anti-CD15^+^ for polymorphonuclear cells (Fig. 3A), (2) anti-CD3^+^ for T lymphocytes (Fig. 3B), (3) anti-CD19^+^ for B lymphocytes (Fig. 3C), and (4) anti-CD14^+^ for monocytes (Fig. 3D). Immunofluorescent staining for WBC subtypes was performed in breast cancer patients (N = 15) and healthy females (N = 15). A positive fluorescent signal from anti-STAU2 was observed in all WBC subtypes. Among the four WBC subtypes, the average fluorescence intensity of anti-STAU2 was significantly increased in CD3^+^ (*p* = 0.0042) and CD19^+^ (*p* = 0.0019) cells. Moreover, the combination of CD3^+^ and CD19^+^, which serves as a marker of the group of T and B lymphocytes, showed a strong increase in the average fluorescence intensity (*p* < 0.0001) (Fig. 3E). The combination of CD3^+^ and CD19^+^ was applied in immunofluorescence staining and fluorescent signal analysis. The blood samples in the coculture model and cell subtype experiment were randomly selected from the whole cohort.

### Fluorescent signal analysis (replicative test)

Then, we performed immunofluorescent staining and obtained the average fluorescence intensity and the percentage of STAU2-positive T and B cells in normal females (N = 363) and breast cancer patients (N = 358). All WBC samples were divided into 10 replicates (sets 1–10) with a double-blinded test. Positive samples from cocultured PBMCs were applied as interassay variation adjustments. Interestingly, a similar pattern of results, which revealed high replicative capacity, was found in all replicates. First, the average fluorescence intensity and the percentage of STAU2-positive cells displayed a linear correlation (R^2^ > 0.9) (Fig. 4A). Second, the average fluorescence intensity of anti-STAU2 in breast cancer patients was significantly increased compared with that in healthy females (*p* < 0.0001) (Fig. 4B). Mammogram (1–3) reports from normal females and breast cancer stage 1–4 patients were obtained to identify potential correlations with the average fluorescence intensity of anti-STAU2. The average fluorescence intensity of anti-STAU2 was not significantly different among WBCs obtained from normal females with 1 to 3 mammograms, but an increasing trend was noted from early-stage to late-stage breast cancer patients (*p* = 0.0008) (Fig. 4C).

### Cut-off point evaluation (sensitivity and specificity)

**Figure 5:**
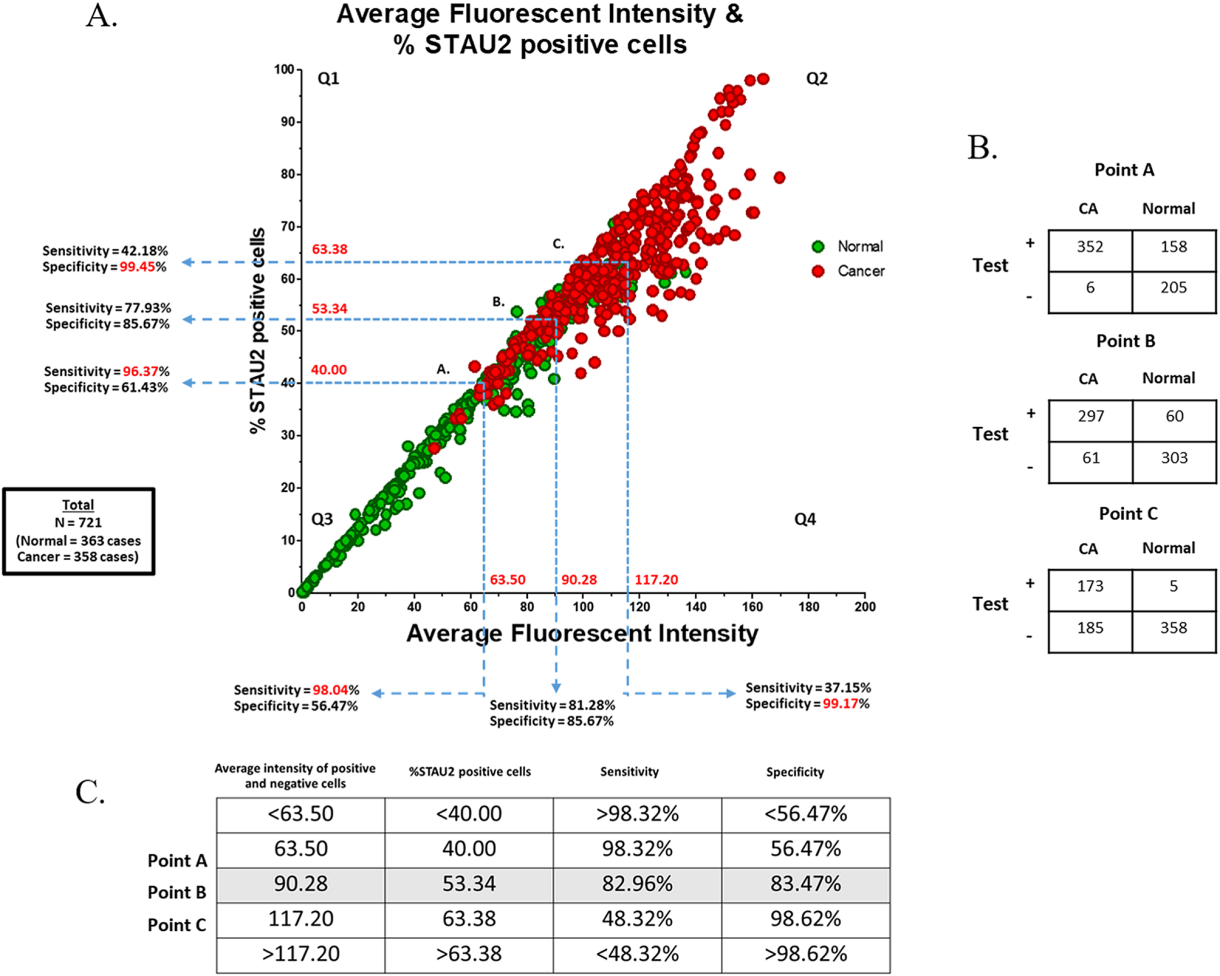
The fluorescent signal of STAU2 in all samples (N = 721) for cut-off point evaluation. (**A**) Three cut-off points (A, B, C) were evaluated. High sensitivity (82.96%) and high specificity (83.47%) were noted at cut-off point B. The intensity detection and the percentage of STAU2-positive cells were operated by CellSens imaging software (Olympus Co., Ltd., USA). (**B**) The 2 × 2 table of positive and negative cases based on cut-off points A, B and C for sensitivity and specificity calculation. (**C**) The sensitivity and specificity details for cut-off points A, B and C showed that cut-off point B was the balanced cut-off point (*p* = 3.56 × 10^–71^, odds ratio = 24.59, 95% CI = 16.64–36.34) with low false positive and negative rates (high accuracy = 83.22%, NPV = 0.8324, PPV = 0.8319).

The average fluorescence intensity and the percentage of STAU2-positive cells in all samples are summarized in Fig. 5A (N = 721). To distinguish normal controls and breast cancer patients, receiver operating characteristic (ROC) curve analysis was performed, revealing the following values: the average fluorescent intensity, 63.50 (Sensitivity = 98.04%, Specificity = 56.47%, NPV = 0.9670, PPV = 0.6896), 90.28 (Sensitivity = 81.28%, Specificity = 85.67%, NPV = 0.8223, PPV = 0.8459), 117.20 (Sensitivity = 37.15%, Specificity = 99.17%, NPV = 0.6154, PPV = 0.9779) and the percentage of positive cells, 40.00 (Sensitivity = 96.37%, Specificity = 61.43%, NPV = 0.9526, PPV = 0.7096), 53.34 (Sensitivity = 77.93%, Specificity = 85.67%, NPV = 0.7974, PPV = 0.8429), 63.38 (Sensitivity = 42.18%, Specificity = 99.45%, NPV = 0.6356, PPV = 0.9869) (Fig. 5A and Supplementary data 5). The combination of the average fluorescence intensity (X-axis) and the percentage of positive cells (Y-axis) was applied as cut-off points, including A (X = 63.50, Y = 40.00), B (X = 90.28, Y = 53.34) and C (X = 117.20, Y = 63.38) (Fig. 5A). The data were separated into 4 quarters (Q1–Q4), including positive (Q1, Q2, Q4) and negative (Q3) areas. The positive and negative cases based on cut-off points A, B and C were counted and classified in a 2 × 2 table (Fig. 5B). The details of the sensitivity and specificity for cut-off points A, B and C are described in Fig. 5C. The sensitivity of the test (98.32%, 82.96% and 48.32%) and specificity (56.47%, 83.47% and 98.62%) were correlated with the percentage of STAU2-positive cells (40, 53.34 and 63.38, respectively). The highest sensitivity (98.32%) was found at cut-off point A (NPV = 0.9716, PPV = 0.6902), and the highest specificity (98.62%) was found at cut-off point C (NPV = 0.6593, PPV = 0.9719). Then, balanced sensitivity (82.96%) and specificity (83.47%) were presented at cut-off point B (NPV = 0.8324, PPV = 0.8319). The average fluorescence intensity of anti-STAU2 and the percentage of positive cells in breast cancer patients (110.50 ± 23.38 and 61.87 ± 12.44) were significantly increased compared with those in healthy females (56.47 ± 32.03 and 33.02 ± 18.10) (*p* = 3.56 × 10^–71^, odds ratio = 24.59, 95% CI = 16.64–36.34) at cut-off point B. In addition, all WBC samples (N = 721) were obtained and subjected to double-blind experiments. The results revealed that STAU2 was a potential marker to identify breast cancer samples with high accuracy = 83.22%. Breast cancer prevalence in the United States (15) was applied to calculate the posterior probability of women under 40 years (prevalence = 1 in 220, 0.455%) to determine whether the risk was approximately equal to or greater than the risk of women > 40 years old (prevalence = 4 in 165, 2.42%) at cut-off point B (posterior probability = 2.25%) (Supplementary data 6).

**Figure 6:**
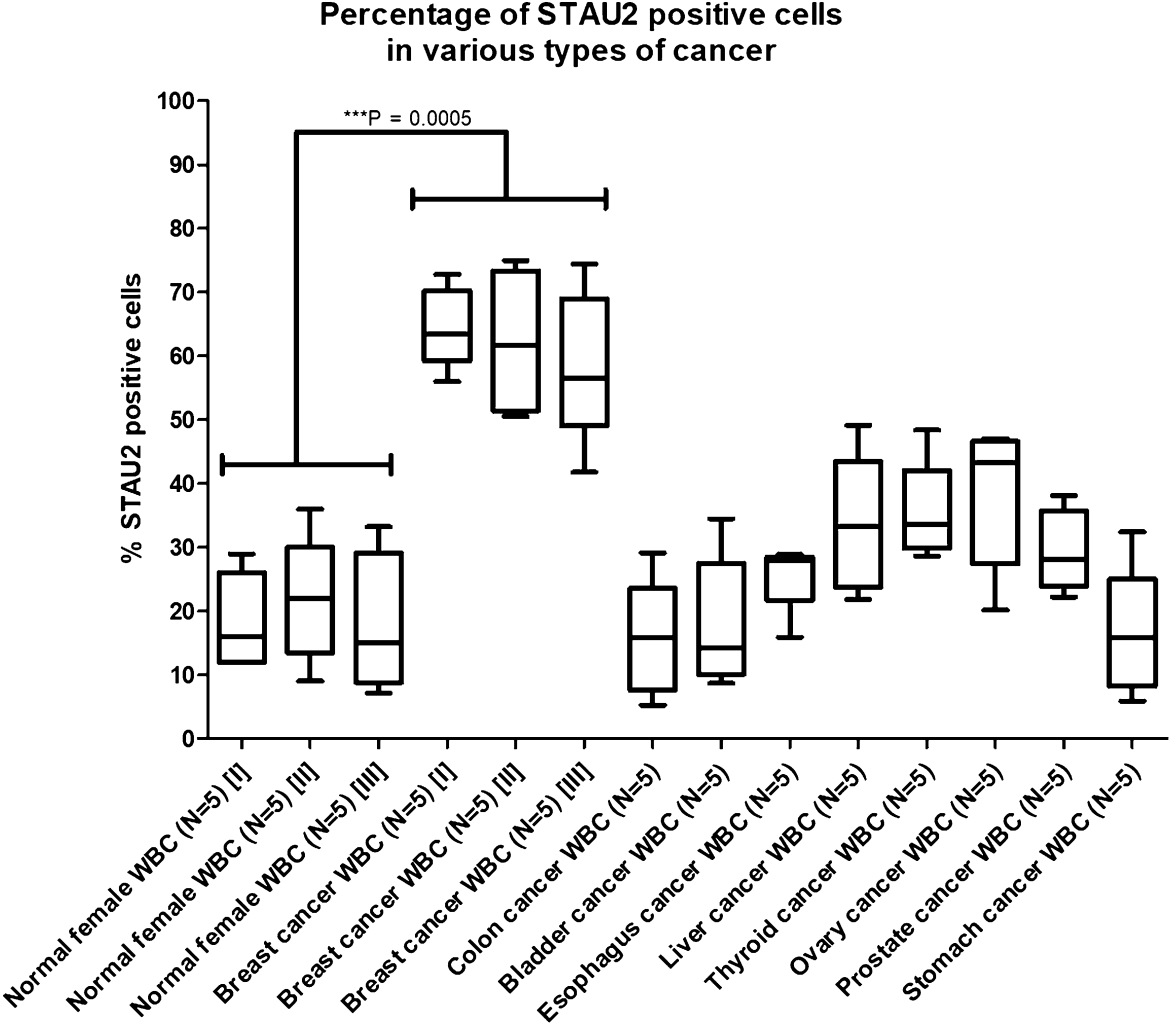
The percentage of fluorescently labelled T and B-cells in various types of cancer, including breast, colon, bladder, oesophageal, liver, thyroid, ovarian, prostate and stomach cancer. The results from normal female and breast cancer samples were randomized into groups I–III. The results demonstrate that STAU2-positive cells were exclusively identified in breast cancer samples (*p* = 0.0005). However, some cancers exhibit a low level of STAU2-positive cells, such as liver, thyroid, ovarian and prostate cancer.

The fluorescent signals from breast cancer T and B cells were analysed and classified based on the type of cellular receptor, including oestrogen receptor (ER), progesterone receptor (PR) and human epidermal growth factor receptor 2 (HER2). The percentage of fluorescently stained cells exhibited a slightly significant increase between the ER+ , PR+ , HER2− and ER+ , PR−, HER2− groups (*p* = 0.0369) (Supplementary data 7). Moreover, to test the specificity of anti-STAU2, high percentages of positive T and B cells were not observed in various cancer types, including colon, bladder, oesophageal, liver, thyroid, ovarian, prostate and stomach cancer. Therefore, STAU2-positive cells were exclusively identified in breast cancer T and B cells (*p* = 0.0005) (Fig. 6). These results reveal that STAU2 can be used as a potential marker in breast cancer screening.

## Discussion

Herein, we report a blood test that is useful for breast cancer screening, particularly for women who are unable to access or have a contraindication for mammography. For example, women under the age of 40 must weigh the risk of radiation exposure with the risk of having breast cancer^37^. The STAU2 blood test can be applied for risk assessment for this group. Supplementary data 6 demonstrates an example of risk calculation and STAU2 results that may indicate further breast imaging tests.

The sensitivity of this tumour marker in T and B cells is high because induction via tumour secretion requires only a few cancer cells. However, the percentages of STAU2 in T and B cells were minimally different among different breast cancer subtypes and stages. Therefore, STAU2 provides minimal benefit for tumour classification and prognosis prediction.

One crucial concern for most patients was residual cancer or recurrence after treatment. Because STAU2 in T and B cells is positive even with a low number of cancer cells, monitoring treatment outcomes using the test will provide information on whether the patients are completely cured from cancer. Nevertheless, the potential use of STAU2 in T and B cells for treatment monitoring requires further research. The secretion of breast cancer-related cells may be distributed throughout the entire body, including bone marrow, and information on the half-life of STAU2 in T and B cells after treatment is not currently available.

WBC protein detection by immunofluorescence techniques provides at least two advantages. First, the technique can distinguish cell types and the intensity of each cell. This information may benefit future diagnosis applications. In addition, the technique is noninvasive, and fixed WBCs in 96-well plates can be transported at room temperature via mail. This benefit makes STAU2 in WBCs a promising biomarker for breast cancer screening public health programmes, including populations with limited resources and radiology experts.

The specific upregulation of STAU2 in WBCs in breast cancer makes the protein a potential target for breast cancer immunotherapy. STAU2 is a staufen double-stranded RNA-binding protein 2, and its gene is located on chromosome 8q21. This protein can interact with zinc finger protein 346 (ZNF346)^38^, which can bind to double-stranded RNA molecules. STAU2 can form a complex with DICER1 and UPF1 RNA helicase^39^ that may be involved in RNA degradation processes and mRNA and protein transport; however, the function of STAU2 protein remains unclear^40^. The upregulated levels of STAU2 protein only in T and B cells might be related to the RNA transport mechanism to produce various inflammatory cytokine (IL-10, TNF-α) molecules that are required to promote tumour growth in the cancer microenvironment^41,42^. Some studies have reported that STAU2 is an anti-apoptotic protein involved in DNA replication and maintenance of genome integrity^43^.

## Conclusion

Our study identified STAU2 protein as a novel breast cancer marker in T and B cells. The quantitative nature of the test provides useful information for risk assessment for a large number of individuals for whom mammograms are not feasible. The other advantages of the use of a cancer marker from a blood sample include its noninvasive nature, ease of accessibility and sample transportation. Therefore, the STAU2 protein in T and B cells represents a promising breast cancer marker for improving breast cancer screening programmes.

## Supplementary Information

Supplementary Information.

## Abbreviations

WBCs: White blood cells
MRI: Magnetic resonance imaging
CTCs: Circulating tumour cells
LINE-1: Long interspersed element 1
MUC-1: Mucin 1
NCBI: National Center for Biotechnology Information
GEO: Gene Expression Omnibus
CU-DREAM: Connection Up- or Downregulation Expression Analysis of Microarrays
STAU2: Staufen protein 2
PBMCs: Peripheral mononuclear cells
DCIS: Ductal carcinoma in situ
CI: Confidence interval
ROC: Receiver operating characteristic
ER: Oestrogen receptor
PR: Progesterone receptor
HER2: Human epidermal growth factor receptor 2
