# Supplementary material for: Quantitative STAU2 measurement in lymphocytes for breast cancer risk assessment
Source: Sci Rep. 2021 Jan 13;11:915. doi: 10.1038/s41598-020-79622-2 (PMC7806934; doi:10.1038/s41598-020-79622-2)

Quantitative STAU2 measurement in lymphocytes for breast cancer risk assessment

Charoenchai Puttipanyalears[1], Sikrit Denariyakoon[2], Phonthep Angsuwatcharakon[1], Vitavat Aksornkitti[1], Mawin Vongsaisuwan[3], Sutasinee Asayut[4], Somchai Thanasitthichai[4], Narisorn Kongruttanachok[5], Chatchawit Aporntewan[1, 6], Apiwat Mutirangura[1*]

[1]*Center of Excellence in Molecular Genetics of Cancer and Human Diseases, Department of Anatomy, Faculty of Medicine, Chulalongkorn University, Bangkok 10330, Thailand*

[2]*The Queen Sirikit Center for Breast Cancer, King Chulalongkorn Memorial Hospital, The Thai Red Cross Society, Bangkok 10330, Thailand*

[3]*Department of Surgery, Faculty of Medicine, Chulalongkorn University, Bangkok 10330, Thailand*

[4]*Research and Technology Assessment Department, National Cancer Institute, Bangkok 10400, Thailand*

[5]*Department of Laboratory Medicine, Faculty of Medicine, Chulalongkorn University, Bangkok 10330, Thailand*

[6]*Department of Mathematics and Computer Science, Faculty of Science, Chulalongkorn University, Bangkok 10330, Thailand.*

Corresponding Author: Prof. Dr. Apiwat Mutirangura, M.D., Ph.D.

Address: Department of Anatomy, Faculty of Medicine, Chulalongkorn University, 1873 Rama IV Road, Pathumwan, Bangkok, 10330 Thailand.

Telephone and Fax no: +662-256-4000

Email: mapiwat@chula.ac.th

Supplementary data 1. Secretory factors released from cancerous tissue to induce epigenetic and phenotypic changes in WBCs were further assessed for the development of a breast cancer screening marker.

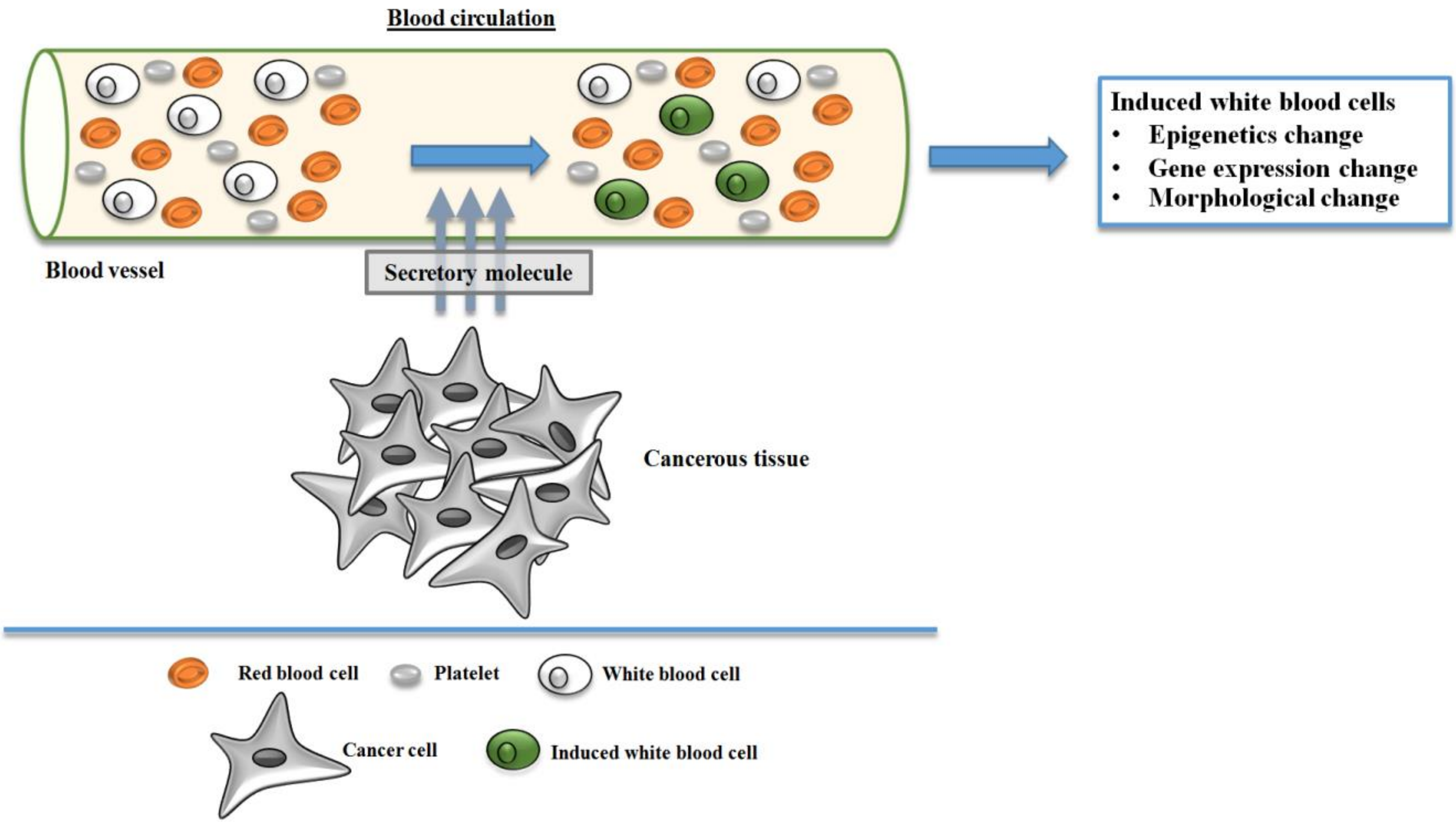

Supplementary data 2. STROBE checklist.

Topic	Item No.	Detail
Title and Abstract	1	Quantitative STAU2 measurement in lymphocytes for breast cancer risk assessment
**Introduction**		
Background/rational	2	Currently, mammography is a widely available population-based technique to detect breast cancer at an early stage. The most effective techniques for breast cancer diagnosis are breast magnetic resonance imaging (MRI), which is also known as MRI mammography, and tissue biopsy, which exhibit >92% sensitivity and specificity. Mammography screening has been sub-optimal accessible in many populations even a population of female health-care workers. It is difficult to set up accessible mammography to general population. Both routine mammography and MRI mammography are costly and require a lot of radiology facilities, leading to inaccessibility for many patients, and tissue biopsy is typically invasive. Recent studies demonstrated that changes in WBCs of cancer patients exhibit promising sensitivity. Accessibility is a key of success in nationwide screening program. To be able to transfer samples by mail has been well known effective approach for mass screening for inborn errors of metabolism
Objectives	3	This study will apply the knowledge of secretion factors on induced WBC changes in blood circulation as a cancer-screening tool. We aimed to develop a blood test using immunofluorescence on fixed WBCs slide to detect cancer-promoting proteins in circulating WBCs of breast cancer patients.
**Methods**		
Study design	4	Three Gene Expression Omnibus (GEO) datasets were collected from the National Center for Biotechnology Information (NCBI) database including GSE9014, GSE31138 and GSE27562. All up-regulated genes containing LINE-1 from GSE9014 and up-regulated genes from GSE31138 & GSE27562 were analyzed, and protein expression was compared using Protein Atlas database. Five candidate genes were selected from the list of up-regulated genes to observe gene expression by immunofluorescence staining in blood samples.
Setting	5	Two ml of EDTA blood samples were collected from 358 breast cancer patients and 363 healthy females between September 2016 and March 2018. All cancer cases were staged according to the revised TNM classification criteria by a pathologist.
Participants	6	All healthy females were recruited from patients without immune disorders, chronic diseases, family history of cancer and negative mammography examination results. The mammogram results of all participants were graded by a radiologist.
Variables	7	The positive fluorescent signals were detected and then classified into the average fluorescent intensity and the percentage of STAU2 positiv cells.
Data sources/ measurement	8	The fluorescent signal from the cells was observed with a confocal microscope (20x) and Motorized fluorescent microscope Type IX83 (Olympus)
Bias	9	Breast cancer WBCs (N=358) and normal female WBCs (N=363) were obtained to perform a double-blinded test in arious cell subtypes including polymorphonuclear cell, monocyte, T lymphocyte and B lymphocyte. The results revealed that STAU2 signal in T and B lymphocytes was a potential marker to identify the breast cancer samples with high accuracy=83.22%.
Study size	10	After obtain the preliminary result, sample size will be calculated with following formula:<br>$n = [(Z_{\alpha/2} + Z_\beta)^2 \sigma^2_d] / \overline{X}_d^2$<br>n = Sample size<br>d = Different of value in each group<br>$\overline{X}_d$ = Different of mean in each group<br>σ^2_d = Different of variance in each group<br>Z = Standard value from normal distribution<br>$Z_{\alpha/2}$ = 1.96<br>Z_β = 1.28<br>SD_d = 8.76<br>$\bar{X}_d$ = 16.48<br>$n = [(Z_{\alpha/2} + Z_\beta)^2 \sigma^2_d] / \bar{X}_d^2$<br>$n = [(1.96 + 1.28)^2 * 8.76^2] / 16.48^2$<br>$n = 2.97$
Quantitative variables	11	The positive cells were shown red and green signal in the same position and the negative cell were shown only red signal. The signal with 25-200 µm perimeter and 140-300 mean gray intensity value were counted.The average fluorescent intensity of Anti-STAU2 in each samples were calculated from the intensity of positive cells divided by the intensity of positive and negative cells. Moreover, the percentage of STAU2 positive cells were calculated by the number of positive cells divided by total number of cells.
Statistical methods	12	Statistical analyses were performed using SPSS (Statistical Package for the Social Sciences) software for Windows version 17.0.1 (SPSS Inc., Chicago, IL). Data are expressed as average ± SD, and independent sample t-tests were performed to calculate significant differences among all sample groups. All p-values are two-sided, and p-values less than 0.05 are considered statistically significant.
**Results**		
Participants	13	WBCs from breast cancer patients (N=358) and healthy females (N=363) were performed immunofluorescence staining. Fluorescent signal from the antibody that binds to a candidate breast cancer-screening marker (STAU2) exhibits signal intensity staining in 25-200 µm perimeter and 140-300 mean gray intensity value. This signal was exclusively observed in breast cancer WBCs. Mammogram (1-3) reports from normal females and breast cancer stage 1-4 patients were obtained to identify potential correlations with the fluorscent intensity and the percentage of STAU2-positive cells.
Descriptive data	14	The intensity and the percentage of STAU2-positive T and B-cells was not significantly different among WBCs obtained from normal females with 1 to 3 mammograms, but an increasing trend is noted from early stage to late stage breast cancer patients (p=0.0008).
Outcome data	15	To distinguish normal controls and breast cancer patients, receiver operating characteristic (ROC) curve analysis was performed, revealing the following values: NPV = 0.8324, PPV = 0.8319, sensitivity=82.96% and specificity=83.47%.
Main results	16	The average fluorescent intensity of Anti-STAU2 and the percentage of positive T and B-cells in breast cancer patients (110.50±23.38 and 61.87±12.44) were significantly increased compared with healthy females (56.47±32.03 and 33.02±18.10) (p=3.56x10-71, Odd Ratio=24.59, 95% CI=16.64-36.34) at cut-off point B. In addition, all WBCs samples (N=721) were obtained and performed experiment in double-blinded test. The results revealed that STAU2 was a potential marker to identify the breast cancer samples with high accuracy = 83.22%.
Other analyses	17	STAU2 is present in all WBC types of all stages and sub-types of breast cancer patients. STAU2 was also found in mononuclear cells of healthy individuals which were co-cultured with breast cancer cell lines.
**Discussion**		
Key results	18	Here, we employed immunofluorescence detection of STAU2 in T and B-cells from 363 healthy females and 358 females with breast cancer. The average fluorescent intensity of Anti-STAU2 and the percentage of positive T and B-cells in breast cancer patients (110.50±23.38 and 61.87±12.44) were significantly increased compared with healthy females (56.47±32.03 and 33.02±18.10) (p=3.56x10-71, Odd Ratio=24.59, 95% CI=16.64-36.34) at cut-off point B.
Limitations	19	The percentages of STAU2 in T and B-cells were minimally different among different breast cancer subtypes and stages. Therefore, STAU2 will provide little benefit for tumor classification and prognosis prediction. The potential use of STAU2 in T and B-cells for treatment monitoring requires further research.
Interpretation	20	The sensitivity of this tumor marker in WBC is high because induction via tumor secretion requires only a few cancer cells. Secretion of breast cancer-related cells may be distributed throughout the entire body, including bone marrow, and information on the half-life of STAU2 in WBCs after treatment is not currently available.
Generalisability	21	WBC protein detection by immunofluorescence techniques provides at least two advantages. First, the technique can distinguish cell types and the intensity of each cell. This information may benefit future diagnosis applications. In addition, the technique is non-invasive, and fixed WBC slides can be transferred via mail. This benefit makes STAU2 in T and B-cells a promising biomarker for breast cancer screening public health programs, including those populations with limited resources and radiology experts.
**Other information**		
Funding	22	This study was financial supported by National Research Council of Thailand (NRCT), Grant number DPG5980005 from Thailand Research Fund (TRF), Ratchadapiseksomphot Fund for Postdoctoral Fellowship, Chulalongkorn University and the Anantara Siam Bangkok Hotel in conjunction with the Four Seasons Hotel Care for Cancer 2017 Fun Run in coordination with the Thai Red Cross Society and Chulalongkorn University. The authors have no other relevant affiliations or financial involvement with any organization or entity with a financial interest in or financial conflict with the subject matter or material discussed in the manuscript apart from those disclosed.

Supplementary data 3. Bioinformatics results from the CU-DREAM and CU-DREAM-extra programs show the intersection results of upregulated genes between GSE9014 and the list of genes containing LINE-1 and the intersection results of upregulated genes between GSE31138 and GSE27562. The list of upregulated genes from both intersections was analysed to identify candidate markers.

Upregulating gene													
GSE 9014													
709 genes													
AKT3	CLEC5A	TMEM108	GUCY1A2	LNX1	LPA	RB1	HMCN1	PHEX	INADL	ATPBD4	KIAA1128	DTNBP1	EVC
ARHGAP24	MC2R	TSGA10	ST6GALNAC3	SLC24A2	SYT14	ZFYVE9	ABHD6	PALM2	GRID1	C8orf34	GPR149	NHS	ATP8B4
NRXN1	PGDS	AGPAT4	LOXL2	SEC24D	SAMD9	POU6F2	ACVR1C	TBC1D5	MED12L	SLC39A9	ADAMTSL1	KCNN2	FLJ32955
HPSE2	CMYA5	DTNA	SHC3	BTNL8	IQCA	DDX24	TMEM135	RPS6KA2	FLJ25371	AZI2	ADAMTSL3	SCHIP1	PCDH15
PLG	ZNF462	OPCML	SLC8A1	KCNH8	FAM19A2	RBMS3	TSHR	IPMK	STAB2	GBP7	GRIK2	TMTC2	FANK1
MMP16	ACCN1	CLASP2	ST5	MMP9	ELP4	JARID1A	CTNND2	CNTNAP5	AFF2	PRICKLE2	CAPSL	C15orf33	COL21A1
NRXN3	KCNAB1	HDAC9	NOX4	SPAST	C20orf39	INPP4B	FAM13A1	C21orf34	BCORL2	PREP	MBOAT2	RGSL1	MS4A5
VAV3	PDGFRA	TASP1	HERC2	CAMTA1	LASS3	ST8SIA5	P4HA3	STK31	FGD3	ASTN2	GRM8	ADAM12	C7orf10
EDIL3	C1orf125	KLHL4	TTC3	KCNJ3	ATP8A2	FGF14	EHBP1	PYGB	SH2D4B	USP15	TFEC	PRMT8	HIVEP1
BAI3	SOX5	GNA12	SPAG17	SPAG6	ARHGAP26	PRDM5	UNC5C	MLZE	XYLB	SGCD	SEC63D1	DBC1	GFRAL
SIRPG	VPS13B	DAPK1	TMEM56	SETBP1	LST-3TM12	GALNT13	CNTNAP2	SYNE1	EPM2A	FBXL7	RYR2	UGT2A1	DSG1
AOAH	FLJ39743	WDR72	PDE10A	ANTXR2	NEB	NPFFR2	ODZ1	FLG	CA10	PGM2L1	ITGA8	LRP1B	SLC41A2
TRA@	C3orf15	LCORL	ADAMTS6	TBC1D8B	ALOX12P2	FCHSD2	RICS	WDR16	FAM5C	FRK	ADAM2	CNKSR2	ANKFN1
ZBTB20	CXorf23	HECW2	LHCGR	CTNNA3	ADAM22	NPHP1	SLC30A7	GLRA2	SUMF1	ABCD2	NXPH1	GDPD4	ERBB4
IL7	CHRM2	KLHL1	APBA1	CREB5	C12orf48	SLC22A3	CTNNA2	MYO6	TINAG	IMPG1	COL4A6	WDR27	TMEM132D
CTAGE5	HTR4	TTBK2	RIMS2	GALNT11	COL4A5	CCDC52	GHR	C12orf4	GRIA3	FRMPD4	ITIH5L	ZAK	CNTN5
IL15	ASCC3	EPS15	CCDC91	MYO1D	RFC3	KIAA1797	MYO3B	KATNAL1	SLC9A11	SIRPD	FAM82A	CYP2C9	PCDH11X
PDE1A	C12orf42	IGSF11	ROBO1	AGXT2	DNAJC10	FLJ20160	CCDC15	KCNMB2	EGF	VPS13C	SLC7A9	MTMR8	NLGN4Y
IL23R	LMO7	ROBO2	PKHD1	PHCA	CSMD2	PLOD1	SYT1	C1orf150	EVI5	ZNF521	C1orf114	USP31	HHLA2
GULP1	ITGA9	TECTA	GJB7	GPR98	KIAA1715	ATRNL1	ST18	NEBL	SCAMP1	DACH2	CXorf59	ABCA13	MICALCL
CFH	GRM7	BNIP3L	VPS54	VPS53	EDA	MAGI3	BACH2	THOC2	RGS6	CUL3	TSHZ2	RTTN	CNTN6
CR1	MRE11A	PRDM11	LRP12	SLC25A24	UACA	KCNQ5	ZNF704	LASS6	ANKHD1	TMEM87A	TTC18	ATP8B1	P2RY14
PDE4D	ZNF644	RAB3IP	ANKRD12	KIF6	PGAP1	NEK1	XKR6	AGTPBP1	C15orf27	PDE1C	CXorf48	KCNIP4	TTC29
CXCL13	FAM49A	USH2A	JMJD2C	FBN2	BOLL	RFX3	GLRA3	TMOD2	ME3	EFHC2	B3GALT1	INVS	KCNB2
ITGB1	ARHGEF7	DAZ2	ARSG	CCDC38	TMC1	GALNT8	PTPRD	BBX	NEK7	FANCC	DAPK2	PTPRT	PACRG
MEF2C	SLC2A13	PRKACB	C12orf28	COL19A1	KCNS3	SLC44A5	BAZ2B	MALL	COL4A4	SH3GL2	DAB1	SLC35F3	NBEA
C9	ABCC9	GAN	MCHR2	SYCP1	ICA1	DPP10	SIL1	CEP192	C12orf40	ADAMTS17	DKFZP564O0823	CNBD1	NOS1AP
CD96	MET	LRRC7	KIAA1432	RIMS1	RXFP1	MDFIC	ARMC4	GPR158	PRLR	RP11-139H14.4	FHL5	SLC16A12	RORA
LILRA2	LPP	ESR1	ARHGAP25	TMEM163	MAPK10	ALK	EPHA3	DGKI	DBT	DKK2	PDE11A	CNGB3	FBXO39
PIK3C2G	PPP1R9A	EPB41L4A	SENP7	WDR52	COL14A1	ANKRD45	PLEKHB2	KCNQ1	C10orf68	LRFN5	GLT8D2	KIFAP3	KIAA1853
SLAMF6	ATP13A4	C14orf145	ITGA2	ATP9B	DNM3	C18orf1	ALDH1A2	GRIK4	GRIA1	TNNI3K	MYRIP	SLC9A2	SEMA3E
CFHR5	MYO9A	PFTK1	ITGBL1	SYT9	SORCS1	RALGPS2	TMEM157	XKR4	KIAA0427	IQCH	LHFPL3	SLC26A7	ADAMTS19
MNDA	TTTY10	CACNA2D3	ANK3	C18orf34	HYDIN	WFDC10B	GPC6	COL8A1	PARP11	MORC4	GPR39	CYP2C18	SPOCK1
FCRL1	DST	PLCXD2	SUSD4	ZNF75	BMPR1B	KIAA1409	STAC	WFDC2	ADAMTS12	FMO5	C9orf126	PRY2	LANCL3
IL1RAPL2	ZRANB3	EPHB1	KIAA0367	HCN1	RYR3	NRG4	DZIP3	THRB	CASP7	SLC15A2	UTX	LOC51057	
CD84	AKAP6	SLC1A1	RFWD2	COBL	PDE4DIP	SLC9A9	SLC38A6	GNA14	PHACTR2	FLJ32549	DISC1	FARS2	
TOX	MUSK	SV2C	COL25A1	COL11A1	C4orf22	LOC401398	NEK11	FANCL	SLC39A12	EMCN	SPOCK3	SH3GL3	
KSR2	CLSTN2	ENPP6	ATP7B	CNOT2	NELL1	C3orf21	TBC1D22A	FLJ40243	BRWD3	MGC24039	TACR3	FAM55D	
MARCO	ZFPM2	HYAL4	RABGAP1L	IQCB1	SGCZ	FSTL5	DIAPH2	CNTN1	GRM5	DIXDC1	FHIT	UTY	
LY96	CNTN4	EPC2	ZNF365	TSPAN5	LYST	NDST3	RASAL2	RASEF	CALN1	C14orf37	CPA6	LRRC28	
ITGAM	PRKG1	NPAL2	EMILIN2	UBL3	NEO1	AUTS2	TLL1	SLC4A4	SCP2	SPATA18	LAMA2	HNT	
C6	SYN2	SH3BGRL2	EEA1	RGL1	CSMD3	CMTM8	EPB41L3	TRPC4	C5orf21	CCDC7	GRIA4	SLC22A15	
NFATC1	COG6	MTMR2	MUC1	RALGPS1	PDE5A	FRAS1	STK32B	F13A1	C1orf9	KLHL20	GPC5	COL22A1	
STAT4	UGT2B17	PCDH11Y	FLJ34870	CSMD1	C9orf68	TMEM16F	CHL1	GSTCD	BMPER	SLC9A10	FAM78B	HERPUD2	
CHIA	AASDH	LOC220115	GRID2	ANKS1B	CAMK2D	CFHR3	MCC	DGKB	POF1B	C3orf35	C1orf101	PPP2R2B	
CYSLTR1	AKAP13	PYHIN1	DKFZp667G2110	CHRM3	METT5D1	GPR83	DENND1B	DDR2	SNAP91	C9orf84	FAM55B	FLJ21511	
ELMO1	PKN2	ANKRD44	CDH12	CNTN3	PPEF1	SPATA5	SNTG1	DNAH3	COL24A1	AKAP7	SV2B	PRKG2	
SPINK5	SLC16A7	DDX58	FAM19A1	UPP2	LOC389634	AR	POFUT2	C13orf26	UTRN	SPON1	CYP2C19	HERC1	
DOCK2	TTC12	DNAH7	ZDHHC21	DMD	ADAMTS20	COG2	ST6GAL2	MAGI2	STAU2	OPRM1	NXF2	FLJ37396	
PDE4B	SCTR	AP3S1	SLC22A16	FNDC3A	RFXDC2	CCDC60	PPP2R3A	DPYD	DOCK4	FBXL13	NCAM1	TRPC5	
PLCH1	DOCK3	L3MBTL4	DLG2	RNF17	HECTD2	PRRG1	DYM	LMAN1	GPR128	SLIT3	MAP2	DLGAP2	
PTGFRN	PCDH19	GPR137B	LSAMP	SMG1	TRDN	LY9	PELI2	IL1RAPL1	ANKRD55	PLSCR2	KCNH1	SORCS2	

Upregulating gene					
Intersect GSE27562 & GSE31138					
262 genes					
STX6	RP1-142L7.9	MARCKS	CDK17	SLMO2	EME1
PHC3	ERBB3	ELAVL1	SMARCA2	EMC7	PLOD1
DENND1B	CMTM6	AZIN1	KLHL24	NUSAP1	
STK38	SUZ12	MPZL1	KLF12	AZI2	
SNX13	TGIF1	PON2	ACO1	SYS1	
BRWD1	C16orf72	B4GALT1	HMMR	CDC42SE1	
C16orf46	TRIM24	RRM2	PTBP3	MEAF6	
SLC5A3	CCP110	UBE2A	PLXNA2	WHSC1L1	
ITGB1	STK4	PLK2	NFATC3	DHX40	
MCM9	ATG7	KPNA1	RAB1A	AACS	
C12orf66	NAPB	CPNE3	CHP1	TMEM33	
RUFY2	TCEB3	IDS	APLP2	TRPS1	
ACTR2	PSME2	DEDD	MSR1	IMPAD1	
CTNNB1	HNRNPD	DLG1	RRAS2	CEP55	
WAPAL	H3F3A	DHFR	SNTB1	CDC73	
GHITM	H3F3AP4	RANBP9	EZR	SDAD1	
PSMD10	H3F3B	GNAQ	ATP6V1E1	PDGFC	
STAU2	TMED2	NRAS	VAPA	CENPU	
DZANK1	ATP6V0E1	GFPT1	TUBB	NETO2	
ABCD3	PRKAR1A	MAP7	ATP2A2	ETNK1	
ZNF678	PFN1	YES1	TUSC3	GPATCH2	
RPRD1A	MARCKSL1	ARFGEF1	TUBA1C	CASD1	
CKAP5	SLC25A5	RHOBTB3	SLC39A8	COA7	
ARMC8	YWHAQ	SSX2IP	RAB27A	SEC61A2	
GCLC	PTP4A1	C5orf15	DCAF7	FJX1	
CAMTA1	GNB1	MTMR2	SLC1A4	CENPN	
NRP2	CALU	PDLIM5	ADAM19	ECT2	
PIAS2	BZW1	LMAN1	METAP2	ASPM	
USP25	RPS15A	SAT1	RIT1	CCDC102B	
RTN4IP1	PAFAH1B1	MRPL19	FAP	PBRM1	
POSTN	SCD	DYNC1LI2	TPX2	BC069739	
RHOA	PRDX6	HELZ	UBE3A	ZNF407	
FAM120A	PSMB1	DLGAP5	IER3IP1	PLA2G12A	
RPUSD3	RAB14	CA12	CDV3	NPL	
CAND1	TAX1BP1	PHACTR2	MCM4	SCYL2	
MESDC1	EIF5B	AURKA	SDC2	C1orf56	
C6orf89	ARF4	TSC22D2	OPA1	MRPS15	
NAA15	USP9X	ENOX2	GRPEL1	DENR	
TBC1D31	PSMA7	WDHD1	NEDD4L	JKAMP	
ZNF654	ACLY	PLK4	CAMSAP1	DTNBP1	
GAB1	PCNA	CCNE2	CSTF2T	SNX12	
NIFK-AS1	TOP2A	RNASE4	NCAPH	CPEB4	
G3BP1	PPIA	SLC25A40	HIPK2	CHMP4B	
HSP90AB1	EIF4A3	ADAM17	HOMER1	CACUL1	
FN1	PLEKHB2	ASPH	COL8A1	ZYG11B	
LPP	ATP6AP2	WASL	RLIM	BTF3L4	
TM2D1	PPIF	PLCL1	C10orf12	ZNF618	
DESI2	ACTG1	ING2	WAC	CISD2	
RRP8	MCM3	NR2C2	NAA50	ZNF827	
C3orf52	OAT	EPHA3	SELT	SPRED1	
ANKH	IER3	PDK3	UBE2J1	STYX	
CLTA	FXR1	G3BP2	TFG	MMP9	

------------->

Candidate gene
DENND1B
STAU2
CAMTA1
LPP
PLEKHB2
MTMR2
LMAN1
PHACTR2
EPHA3
COL8A1
DTNBP1
AZI2
MMP9
PLOD1

	GSE9014				
	Up (0.05)	Not Up			
L1	709	488	1.197	P-value	1.34E-08
No L1	9.524	8.054	17.758	Odd ratio	1.39
	10.233	8.542	18.775		

		GSE31138_up				
		Up (0.01)	Not Up			
GSE27562_Up	Up (0.01)	262	3.178	3.440	P-value	2.62E-65
	Not Up	433	19.123	19.556	Odd ratio	3.64
		695	22.301	22.996		

Supplementary data 4. Immunofluorescence staining results of 4 candidates, including A) anti-LMAN1, B) anti-AZI2, C) anti-MMP9 and D) anti-PLOD1. Hoechst was applied to locate the WBC position. Anti-CD45+ was used as a positive control for WBCs. The immunofluorescence staining of all 4 candidates exhibited no significant fluorescence signals between breast cancer and normal female WBCs.

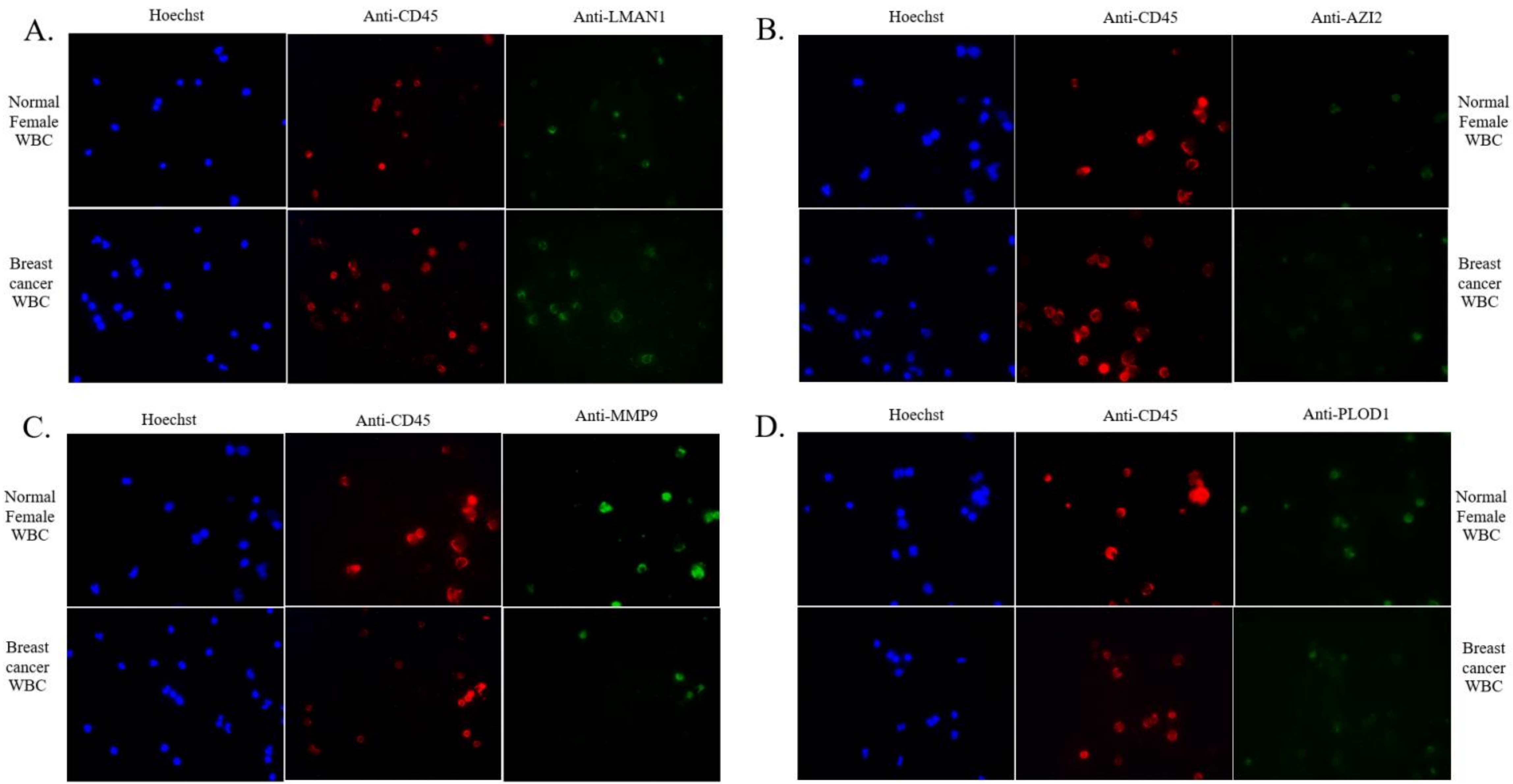

Supplementary data 5. ROC curve analysis was performed in normal female (N=363) and breast cancer (N=358) samples for cut-off point evaluation. A) ROC curve of the average fluorescence intensity (cut-off point 90.28, sensitivity = 81.28%, specificity = 85.67). B) ROC curve of the percentage of positive cells (cut-off point 53.34, sensitivity = 77.93%, specificity = 85.67%). ROC curve analysis was performed using MedCalc (Statistical software) for Windows version 11.3.0.0 (Ostend, Belgium).

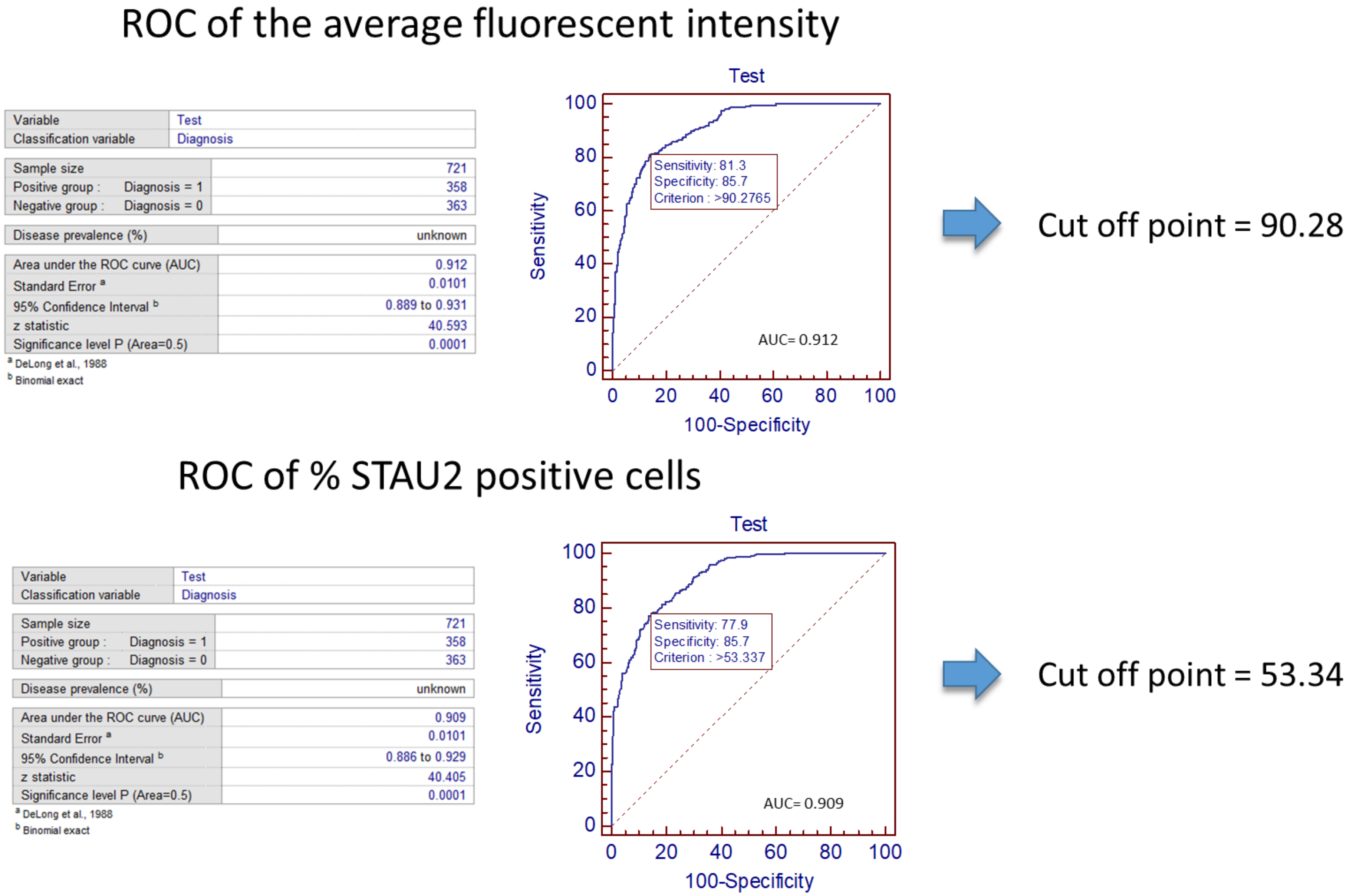

ROC of the average fluorescent intensity

Variable	Test
Classification variable	Diagnosis

Sample size		721
Positive group :	Diagnosis = 1	358
Negative group :	Diagnosis = 0	363

Disease prevalence (%)	unknown

Area under the ROC curve (AUC)	0.912
Standard Error [a]	0.0101
95% Confidence Interval [b]	0.889 to 0.931
z statistic	40.593
Significance level P (Area=0.5)	0.0001

[a] DeLong et al., 1988
[b] Binomial exact

ROC of % STAU2 positive cells

Variable	Test
Classification variable	Diagnosis

Sample size		721
Positive group :	Diagnosis = 1	358
Negative group :	Diagnosis = 0	363

Disease prevalence (%)	unknown

Area under the ROC curve (AUC)	0.909
Standard Error [a]	0.0101
95% Confidence Interval [b]	0.886 to 0.929
z statistic	40.405
Significance level P (Area=0.5)	0.0001

[a] DeLong et al., 1988
[b] Binomial exact

Supplementary data 6. The posterior probability calculation. The breast cancer prevalence in the United States was calculated as the prior probability (P). Condition probability (C) was determined based on sensitivity and false positive rate in each cut-off point. Joint probability (J) in the cancer (JCA = PCA x CCA) and normal (JN = PN x CN) groups was evaluated. The posterior probability (risk) of a positive result being cancer ((JCA/(JCA+ JN)) and normal ((JN/(JCA+ JN)) was calculated using JCA and JN values. The risk of women under 40 years (prevalence = 1 in 220, 0.455%) is approximately equal to or greater than the risk of women >40 years old (prevalence = 1 in 165, 2.42%) at cut-off point B (posterior probability = 2.25%).

		Cancer (CA)	Normal (N)
Prior probability (P)		1/220 = 0.00455 (American Cancer Society. Breast Cancer Facts & Figures 2017-2018. Atlanta: American Cancer Society, Inc. 2017)	219/220 = 0.9955
Condition probability (C)	Point A	352/358 (sensitivity = 98.32%) (352 cases from the area over point A)	158/363 (false positive rate = 0.4353) (158 case from the area over point A)
	Point B	297/358 (sensitivity = 82.96%) (297 cases from the area over point B)	60/363 (false positive rate = 0.1653) (60 case from the area over point B)
	Point C	173/358 (sensitivity = 48.32%) (173 cases from the area over point C)	5/363 (false positive rate = 0.0138) (5 case from the area over point C)
Joint probability (J)		$P_{CA} \times C_{CA} = J_{CA}$	$P_N \times C_N = J_N$
	Point A	$(1/220) \times (352/358) = 4.47\text{x}10^{-3}$	$(219/220) \times (158/363) = 0.433$
	Point B	$(1/220) \times (297/358) = 3.77\text{x}10^{-3}$	$(219/220) \times (60/363) = 0.164$
	Point C	$(1/220) \times (173/358) = 2.20\text{x}10^{-3}$	$(219/220) \times (5/363) = 0.014$

Posterior probability

Positive results can be cancer. $\frac{J_{CA}}{J_{CA}+J_N}$

Positive results can be normal. $\frac{J_N}{J_{CA}+J_N}$

Point A

$$\frac{4.47\text{x}10^{-3}}{(4.47\text{x}10^{-3}) + (0.433)} = 1.07\% \qquad \frac{0.433}{(4.47\text{x}10^{-3}) + (0.433)} = 98.98\%$$

Point B

$$\frac{3.77\text{x}10^{-3}}{(3.77\text{x}10^{-3}) + (0.164)} = 2.25\% \qquad \frac{0.164}{(3.77\text{x}10^{-3}) + (0.164)} = 97.75\%$$

Point C

$$\frac{2.20\text{x}10^{-3}}{(2.20\text{x}10^{-3}) + (0.014)} = 13.58\% \qquad \frac{0.014}{(2.20\text{x}10^{-3}) + (0.014)} = 86.42\%$$

Prevalence of breast cancer in age below 40 = 1 case in 220 = 0.455%

Prevalence of breast cancer in age above 40 = 4 cases in 165 = 2.42%

Supplementary data 7. The percentages of breast cancer T and B cells expressing STAU2 were analysed and classified based on the expression of cellular receptors, including A) oestrogen receptor (ER), progesterone receptor (PR) and human epidermal growth factor receptor 2 (HER2). B) The percentage of fluorescently labelled cells was analysed among cells expressing ER, PR and HER2. The percentage of fluorescently labelled cells exhibited a slightly significant increase among the ER+, PR+, HER2- and ER+, PR-, HER2- groups (p = 0.0369).

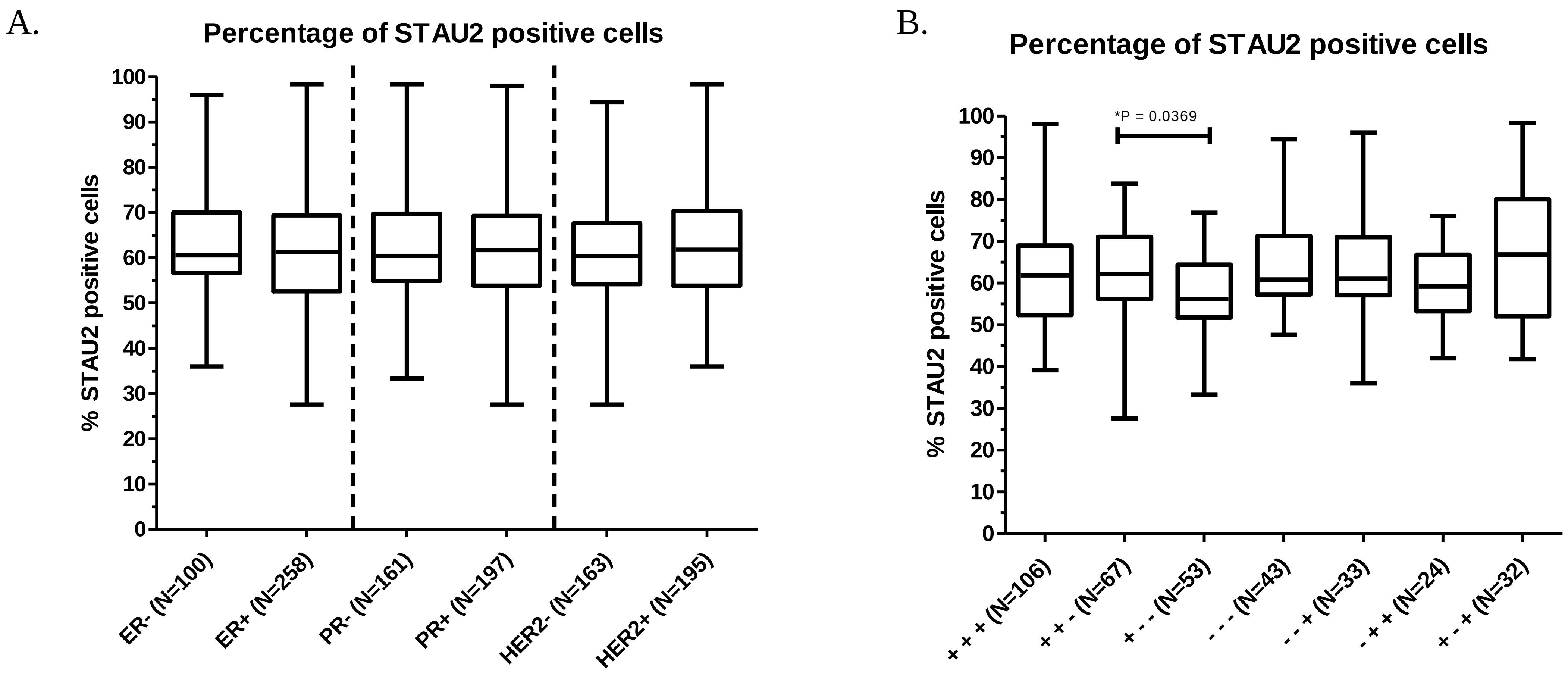

Supplement: Supplementary file 1 — Supplementary Information. [file 41598_2020_79622_MOESM1_ESM.pdf]
